# Summary of Clinical Cases Presented to the Medical Class Attending the Mercy Hospital during the Month of November, 1858

**Published:** 1858-12

**Authors:** E. O. F. Roler


### SUMMARY OF CLINICAL CASES PRESENTED TO THE MEDICAL CLASS ATTENDING THE MERCY HOSPITAL DURING THE MONTH OF NOVEMBER, 1858. BY E. O. F. ROLER.

The number of students attending the wards of the Mercy Hospital for clinical instruction, during the present session of Rush Medical College, is seventy-five. They are divided into two classes; one visiting the Hospital every Monday, Wednesday and Friday, and the other every Tuesday, Thursday and Saturday. The cases which have been fully, and in some instances repeatedly, presented to them during the clinic hours of the last month (November) are as follows:

1. Miss Ellen ——, aged twenty-one years, native of Ireland; has organic disease of the valves of the pulmonary artery with hypertrophy, and also signs of incipient tuberculosis of the apex of the right lung. The cardiac disease is the sequel of an attack of rheumatism three years since. The case is still under treatment.

2. Miss Kate ——, aged twenty years, native of Ireland; had well-marked typhoid fever. Discharged well.

3. Miss Mary ——, aged eighteen years, native of Germany; had a malignant pustule on the thumb. Discharged well.

4. Mrs. Mary ——, aged thirty-four, native of Ireland; had ascites, with an abdominal tumor extending from the left hypochondriac region down to the pubes, and from the concavity of the left ileum to the linea alba. After being under treatment four weeks she left the Hospital much improved.

5. Miss Ann ——, aged twenty-eight years; has chronic gastritis. Still under treatment.

6. Mrs. S. ——, aged forty-seven years, native of Ireland; had scirrhus of the pylorus, accompanied by extreme emaciation and inability to retain either food or medicine without extreme distress. Died.

7. Mrs. Mary ——, aged eighty-two years, native of Ireland; has extensive chronic bronchitis, with much emaciation, and copious purulent expectoration. Still under treatment.

8. Mrs. L. ——, aged forty-five years, native of Germany; had uterine hemorrhage. Recovered.

9. Miss Bridget ——, aged twenty-two years; had menorrhagia. Recovered.

10. Mrs. Bridget ——, aged twenty-three years, native of Ireland; had tubercular phthisis, with extensive cavities, copious purulent expectoration, and extreme emaciation. Died.

11. Mrs. Alice ——, aged thirty years, native of Ireland; has incipient tuberculosis. Still under treatment.

12. Miss Ellie ——, aged seven years; had rubeola. Recovered.

13. Miss Mary ——, aged six years; had phagedenic ulcer on the inner surface of the right cheek, with anemia. Much improved, but still under treatment.

14. Mrs. C. ——, aged forty years, native of Germany; had pneumonia. Recovered.

15. James W., aged thirty-one years, native of Ireland; admitted with complete paraphlegia, accompanied by paralysis of the sphincters of the bladder and rectum, and extensive bed-sores over the sacrum and hips. Diagnosis—disorganization of the spinal cord from sub-acute inflammation, commencing three months since. Died about two weeks after admission.

16. Anton ——, a German, aged thirty-five years; had typhoid pneumonia. Recovered.

17. Timothy C——, native of Ireland, aged thirty-eight years; had simple intermittent fever. Recovered.

18. James Mc——, native of Ireland, aged twenty-seven years; had acute conjunctivitis. Recovered.

19. Dennis H——, native of Ireland, aged thirty-two years; had pernicious intermittent, with symptoms strongly marked. Recovered.

20. Barney D——, aged twenty-six years; had typhoid fever. Recovered.

21. J. McM——, native of Ireland, aged forty-six years; had tubercular phthisis in the third stage of advancement. Still under treatment.

22. Peter ——, native of Germany, aged fifteen years; had scarlatina anginosa. Recovered.

23. John ——, aged twenty-nine years; had tubercular phthisis—its last stage. Died.

24. Moretz ——, a native of Germany, aged thirty years; had tubercular phthisis in its last stage. Died.

25. Antony D——, aged twenty-six years; had chronic bronchitis. Left the Hospital much improved.

26. John ——, a native of Germany, aged sixty-three years; had sub-acute laryngitis. Recovered.

27. Eugene ——, native of France, aged thirty-three years; had chronic dysentery with ascites. Still under treatment.

28. Charles B——, aged twenty-two years; had chronic dysentery. Discharged well.

29. James C——, native of Germany, aged twenty-one years; had complete paraphlegia, including partial paralysis of the bladder and rectum. Attack was developed rapidly and without fever or any appreciable cause. Left the Hospital with recovery nearly complete.

30. Patrick ——, native of Ireland, aged thirty-seven years; had chronic conjunctivitis, with partial opacity of the cornea. Improved.

31. Edward B——, aged twenty-three years; had chronic bronchitis. Recovered.

32. James C——, aged seven years; had simple continued fever. Recovered.

33. James ——, aged eight years; had rubeola. Recovered.

34. Charles V——, aged four years; had impetigo. Recovered.

35. Patrick S——, aged eight years; had porrigo scutulata. Recovered.

36. John ——, aged seven years; had scrofulous swelling of the glands of the neck. Still under treatment.

37. Michael McG——, a native of Ireland, aged thirty-six yeors; had duodinitis with jaundice. Still under treatment.

38. David K——, aged twenty-three years; had typhoid fever. Recovered.

39. Karl B——, a German, aged thirty-one years; had eczema. Recovered.

40. James D——, aged eight years; had corneitis. Still under treatment.

During the daily clinics, whenever the cases under consideration presented cardiac or pectoral symptoms, each member of the class was required to practice auscultation, and examine the patient in such a manner as to acquire practical tact in diagnosis.

